# Perceived organizational toxicity and workplace loneliness among public-school teachers in one province of Türkiye: a cross-sectional statistical mediation analysis of organizational agility

**DOI:** 10.3389/fpsyg.2026.1916512

**Published:** 2026-08-06

**Authors:** Ahmet Mesut Karadağ, Gökhan Kahveci

**Affiliations:** 1 Department of Educational Sciences, Institute of Graduate Education, Recep Tayyip Erdoğan University, Rize, Türkiye; 2 Department of Educational Sciences, Faculty of Education, Recep Tayyip Erdoğan University, Rize, Türkiye

**Keywords:** mediation, organizational agility, organizational psychology, organizational toxicity, school climate, teachers, Türkiye, workplace loneliness

## Abstract

**Introduction:**

Teachers’ work is closely connected with communication, collegial support, coordination, and trust within the school environment. When these relationships are not experienced as sufficiently supportive or satisfying, teachers may experience loneliness at work. This study examined the associations among teachers’ perceived organizational toxicity, organizational agility, and workplace loneliness, and tested whether organizational agility statistically mediated the association between organizational toxicity and workplace loneliness.

**Methods:**

A cross-sectional correlational design was used. Data were collected through self-report scales from 301 teachers working in public primary and lower-secondary schools in the central district of Rize Province, Türkiye. The data were analyzed using descriptive statistics, correlation analysis, confirmatory factor analysis, structural equation modeling, and bootstrap analysis based on 5,000 resamples.

**Results:**

Teachers reported descriptively low levels of perceived organizational toxicity and workplace loneliness and a high level of organizational agility. Perceived organizational toxicity was negatively associated with organizational agility and positively associated with workplace loneliness, whereas organizational agility was negatively associated with workplace loneliness. In the structural model, the direct association between perceived organizational toxicity and workplace loneliness was positive and statistically significant (*β* = 0.444). The indirect association through organizational agility was also statistically supported (*B* = 0.097, *β* = 0.134, 95% bias-corrected bootstrap CI for B [0.042, 0.164]). Because the direct association remained statistically significant, the findings supported a pattern of partial statistical mediation. However, a reverse-order alternative model produced identical global fit, indicating that the cross-sectional data could not establish the temporal or causal ordering of the variables.

**Discussion:**

The findings suggest that teachers’ workplace loneliness may be considered in relation to both the relational climate and the functional response capacity of schools. Given the cross-sectional and self-report design, the results should be interpreted as statistical associations rather than causal effects.

## 1 Introduction

Teaching is not limited to instructional activities carried out in the classroom. Teachers’ daily work is shaped through continuous interaction with students, colleagues, school administrators, and families. In this respect, the school is both a pedagogical and a social workplace. However, the fact that schools are interaction-rich environments does not necessarily mean that teachers always feel supported, understood, or professionally connected. Workplace loneliness refers to employees’ subjective perception that the social relationships they need in the workplace are not sufficiently available or do not provide the expected level of satisfaction (Wright et al., 2006). In the school context, this experience is important because teachers’ professional lives often gain meaning through sharing, trust, emotional support, collaborative problem solving, and a sense of belonging to the school community.

Research on workplace loneliness indicates that this experience is relevant to both individual well-being and organizational life. In studies with teachers, workplace loneliness has been associated with life satisfaction (Yılmaz and Arslan, 2013). In broader employee samples, workplace loneliness has been examined in relation to absenteeism, burnout, job performance, job satisfaction, turnover intention, and well-being-related outcomes (Bowers et al., 2022; Bryan et al., 2023; Erdil and Ertosun, 2011; Özçelik and Barsade, 2018; Sasaki et al., 2025). These studies suggest that loneliness cannot be explained only through individual characteristics; the quality of workplace relationships, available support, and organizational conditions may also be important for understanding this experience.

One organizational condition that may be considered together with teachers’ workplace loneliness is perceived organizational toxicity. Toxic workplace environments have been discussed in relation to negative interaction patterns, employee strain, organizational support, well-being, and work-related outcomes (Rasool et al., 2019, 2021; Wang et al., 2020). Such environments may include aggression, bullying, disrespect, exclusion, humiliation, intimidation, or similar behaviors that make constructive communication more difficult (Rasool et al., 2021; Wang et al., 2020). Studies on workplace incivility also show that relatively low-intensity disrespectful behaviors may become more wearing when they are repeated or sustained through reciprocal negative reactions (Andersson and Pearson, 1999; Ghosh et al., 2011). In educational organizations, toxic leadership has been addressed through five dimensions: abusive supervision, authoritarian leadership, narcissism, self-aggrandizement, and unpredictability (Konan and Kırbaç, 2023). The relationship between school principals’ toxic leadership behaviors and teachers’ perceived stress has also been examined in the educational context (Balaban and Kazancı-Tınmaz, 2024).

A toxic school environment should not be reduced only to the negative behaviors of particular individuals. Routines, informal rules, and inconsistent ways of functioning that make it difficult to discuss problems openly may also become part of this atmosphere over time (Van Rooij and Fine, 2018). In such an environment, teachers may become more cautious when sharing professional concerns, avoid seeking support, or withdraw from everyday collegial interactions. Given the cross-sectional nature of the present study, this interpretation should not be read as a causal explanation, but as a theoretically meaningful association. It is nevertheless consistent with the view that loneliness is related less to the number of people around an individual than to the perceived quality of social relationships (Erdil and Ertosun, 2011; Hawkley and Cacioppo, 2010; Wright et al., 2006).

Another organizational characteristic considered in this study is organizational agility. Organizational agility is generally defined as an organization’s capacity to notice changes, respond to emerging needs, and adapt its functioning in a timely manner (Overby et al., 2006; Sharifi and Zhang, 1999). Previous studies have explained agility through characteristics such as adaptability, flexibility, responsiveness, resource reconfiguration, and the ability to act appropriately under changing conditions (Asghar et al., 2026; Doz and Kosonen, 2010; Mrugalska and Ahmed, 2021; Sambamurthy et al., 2003; Sherehiy et al., 2007; Tallon and Pinsonneault, 2011; Teece et al., 2016). Measurement and adaptation studies also indicate that organizational agility can be assessed through employees’ organizational perceptions and can be treated as a measurable construct in Turkish samples (Akkaya and Tabak, 2018; Gürbüz and Hatunoğlu, 2022).

In the school context, organizational agility is examined in this study through teachers’ perceptions of the school’s capacity to respond to changing conditions and emerging needs. At this point, agility should not be viewed as a general characteristic that produces positive outcomes under all circumstances. The relationship between agility and organizational outcomes may vary depending on contextual and accompanying organizational conditions (Bekos et al., 2025). For this reason, the present study positions organizational agility as an organizational perception that may help explain the association between perceived organizational toxicity and workplace loneliness.

Research on workplace loneliness, toxic work environments, and organizational agility provides an important basis for understanding teachers’ school experiences. Yet it remains insufficiently clear how these three constructs are related to one another in the school context. Workplace loneliness has often been examined in general employee samples, whereas teachers’ loneliness experiences have been studied more limitedly in relation to school-level organizational characteristics. Toxic workplace environments have been associated with stress, well-being, organizational support, and work-related outcomes, but the association between perceived organizational toxicity and workplace loneliness still requires further explanation in schools. Similarly, organizational agility has mostly been discussed as a capacity for adaptation and responsiveness, while its relationship with teachers’ social and psychological work experiences has been addressed less directly.

The theoretical contribution of this study lies in discussing teachers’ workplace loneliness not only through individual characteristics or directly relational variables, but also through an organizational perception related to the school’s functional response capacity. The workplace loneliness literature shows that loneliness is associated with work-related variables such as employee well-being, job satisfaction, burnout, performance, and managerial relationships. However, because a considerable part of this literature is cross-sectional, the direction of these associations should be interpreted with caution (Bryan et al., 2023; Özçelik and Barsade, 2018). Therefore, when explaining teachers’ loneliness experiences, it may be useful to consider not only more proximal relational constructs, such as perceived organizational support, trust, or psychological safety, but also broader aspects of school functioning, such as how schools notice problems, circulate information, and respond to changing needs (Edmondson, 1999; Newman et al., 2017; Frazier et al., 2017; Tian et al., 2023).

In this context, organizational agility is not treated merely as the school’s capacity to make quick decisions. Rather, it is considered as teachers’ perception of the school’s capacity to notice changing conditions, develop appropriate responses to emerging needs, adapt resources and routines to the situation, and maintain coordination when conditions change (Sharifi and Zhang, 1999; Overby et al., 2006; Teece et al., 2016). Recent research also suggests that organizational agility should not be reduced to a single feature such as quickness or flexibility, but should be considered as a multidimensional construct related to contextual factors, fundamental attributes, enabling mechanisms, and dynamic capabilities (Asghar et al., 2026). For this reason, the present study does not position organizational agility as a substitute for more proximal relational constructs such as perceived organizational support, trust, or psychological safety. Instead, it positions organizational agility as a complementary organizational mechanism that may help explain the association between a toxic school perception and teachers’ workplace loneliness at the level of the school’s functional response capacity.

Within this framework, the present study examines the associations among teachers’ perceived organizational toxicity, organizational agility, and workplace loneliness in public primary and lower-secondary schools in the central district of Rize Province, Türkiye. Organizational agility is positioned as a mediating organizational perception in the association between perceived organizational toxicity and workplace loneliness. Because the study uses a cross-sectional design, the proposed relationships are interpreted as associations among variables rather than causal effects.

## 2 Literature review

### 2.1 Workplace loneliness

Workplace loneliness refers to employees’ subjective evaluation that they cannot establish the social relationships they desire in the workplace at a sufficient or satisfying level. For this reason, loneliness cannot be explained simply by the number of people around an individual. Employees who are in frequent contact with many people in the same work environment may still feel misunderstood, unsupported, or relationally distant. Wright et al. (2006) conceptualized workplace loneliness as a construct related to a lack of social relationships in the work environment and developed a scale to measure it. Hawkley and Cacioppo (2010) also noted that loneliness is related to perceived social isolation and is closely connected with how individuals experience their social surroundings.

At first glance, the school environment may not seem easy to associate with loneliness. Teachers are in contact with students, colleagues, and administrators throughout the day. Yet working in a socially crowded environment does not necessarily mean that teachers feel professionally supported or see themselves as a meaningful part of the school community. Teachers’ experience of workplace loneliness may be considered together with limited professional sharing, weakened trust, the absence of suitable spaces to talk about common problems, or the inability to establish supportive relationships at the expected level. This interpretation is consistent with the view that loneliness is related more to the perceived quality of relationships than to their quantity (Erdil and Ertosun, 2011; Hawkley and Cacioppo, 2010; Wright et al., 2006).

Some studies on teachers suggest that workplace loneliness may be related to teachers’ broader life experiences. Yılmaz and Arslan (2013) examined the relationship between teachers’ workplace loneliness and life satisfaction. Research conducted during the pandemic also showed that loneliness, quality of life, happiness, and similar variables could be examined together among teachers and school administrators (Karakose et al., 2022). In broader employee samples, workplace loneliness has been discussed in relation to absenteeism, job performance, turnover intention, and well-being-related outcomes (Bowers et al., 2022; Erdil and Ertosun, 2011; Özçelik and Barsade, 2018; Sasaki et al., 2025). The systematic review and meta-analysis by Bryan et al. (2023) also emphasized that workplace loneliness may be associated with job satisfaction, burnout, and some performance indicators, while noting that causal interpretations require caution because many studies in the field are cross-sectional.

Within this framework, workplace loneliness should not be viewed only as an individual emotional state for teachers. How relationships are formed at school, how problems are discussed, and to what extent teachers can receive support from colleagues and administrators may all be important for understanding this experience. Considering teachers’ workplace loneliness together with the relational climate and organizational functioning of schools may therefore help clarify the meaning of this construct in the school context.

### 2.2 Organizational toxicity

Organizational toxicity is a concept related to employees’ perceptions of the work environment as wearing, unsafe, exclusionary, or difficult for building healthy relationships. The concept is usually considered not as a single negative behavior, but together with recurring patterns of behavior that weaken the quality of workplace relationships over time. Toxic workplace environments have been examined in relation to employee well-being, stress, perceived organizational support, and some work-related outcomes (Rasool et al., 2019, 2021; Wang et al., 2020). Such environments may include aggression, bullying, disrespect, exclusion, humiliation, intimidation, or similar behaviors that make constructive communication more difficult (Rasool et al., 2021; Wang et al., 2020).

A toxic work environment may not always begin with explicit or severe behaviors. Sometimes lower-intensity forms of disrespect, sarcastic remarks, or humiliating interactions may gradually damage workplace relationships. Andersson and Pearson (1999) described workplace incivility as a process that may escalate through reciprocal reactions. Ghosh et al. (2011) also referred to a continuum extending from incivility to more severe negative behaviors. This perspective makes it possible to consider toxicity not only through clearly aggressive behaviors, but also through smaller ruptures that accumulate in everyday communication.

In the school context, organizational toxicity may be closely related to how teachers experience their work environment. Administrators’ attitudes, communication among colleagues, the way problems are discussed, and informal norms may shape teachers’ perceptions of the school environment. In educational organizations, toxic leadership has been addressed through five dimensions: abusive supervision, authoritarian leadership, narcissism, self-aggrandizement, and unpredictability (Konan and Kırbaç, 2023). The relationship between school principals’ toxic leadership behaviors and teachers’ perceived stress has also been examined (Balaban and Kazancı-Tınmaz, 2024). These findings suggest that toxic school experiences may not remain limited to individual discomfort, but may also be related to how teachers evaluate the school environment and their relationships within it.

It is also important not to reduce toxicity only to individual behaviors. In some cases, the everyday functioning of an organization, habits that make open discussion difficult, or informal rules that lead problems to be ignored may also become part of a negative atmosphere. Van Rooij and Fine (2018) emphasized that organizational processes and ways of functioning that normalize deviance should be considered when evaluating toxic organizational culture. This approach makes it possible to examine toxicity in schools not only through the behavior of particular individuals, but also through how the school approaches problems and organizes communication.

Teachers’ perception of a toxic school environment may be associated not only with workplace loneliness, but also with broader perceptions of how the school functions. Toxic or uncivil interactions have been associated with negative interaction processes that may weaken trust, respect, and willingness to communicate openly among employees (Andersson and Pearson, 1999; Ghosh et al., 2011). The psychological safety literature also suggests that employees’ willingness to express views, share problems, engage in learning behavior, and take interpersonal risks is related to the perceived safety of the social context in which they work (Edmondson, 1999; Newman et al., 2017; Frazier et al., 2017). Similarly, the employee voice literature indicates that speaking up about work-related issues may be associated with managerial openness, perceived safety of voice, and implicit patterns of self-censorship (Detert and Burris, 2007; Detert and Edmondson, 2011). From this perspective, when the school environment is perceived as more toxic, teachers may be less willing to raise problems openly, share information with colleagues or administrators, and participate in joint problem-solving processes. This interpretation should not be read as a causal claim that toxicity directly reduces organizational agility. Rather, it provides a theoretical explanation for why perceived toxicity may be negatively associated with teachers’ perceptions of the school’s information flow, coordination, and timely response capacity.

### 2.3 Organizational agility

Organizational agility is a concept related to an organization’s capacity to notice changing conditions, respond to emerging needs, and adapt its functioning accordingly. Sharifi and Zhang (1999) associated agility with the ability to respond to change and benefit from change. Overby et al. (2006) treated agility as the capacity to sense environmental changes and respond to them. These definitions show that agility is not limited to acting quickly; it also includes recognizing change, developing an appropriate response, and adapting organizational functioning to the situation.

In the agility literature, the concept is generally explained through characteristics such as adaptability, flexibility, responsiveness, quickness, and the ability to reconfigure resources (Asghar et al., 2026; Doz and Kosonen, 2010; Mrugalska and Ahmed, 2021; Sambamurthy et al., 2003; Sherehiy et al., 2007; Tallon and Pinsonneault, 2011; Teece et al., 2016). Sherehiy et al. (2007) showed that agility has been addressed together with multiple characteristics across different studies. Asghar et al. (2026) also indicated that organizational agility continues to be discussed as a multidimensional concept. Therefore, agility can be evaluated not as a single behavior or a single organizational feature, but as a broader perception of how an organization acts under changing conditions.

In educational organizations, agility has gained importance especially in connection with changing expectations, policy regulations, technological developments, and periods of crisis. However, using agility in the school context as if it were a performance concept transferred directly from business organizations requires caution. In schools, agility may gain meaning through teachers’ perceptions of the school’s functioning, response capacity, and way of dealing with changing conditions. Scale adaptation studies in the Turkish literature also indicate that organizational agility can be treated as a measurable construct through employee perceptions (Akkaya and Tabak, 2018; Gürbüz and Hatunoğlu, 2022).

The relationship between organizational agility and employee experiences has received increasing attention in recent years. For example, some studies suggest that employees’ perceptions of organizational agility may be associated with work engagement and well-being (Ludviga and Kalvina, 2024). At the same time, agility should not be treated as a general organizational characteristic that produces positive outcomes under all conditions. The outcomes associated with organizational agility may vary depending on contextual conditions, complementary capabilities, and how agility is experienced by employees (Bekos et al., 2025). Therefore, in the school context, agility should not be understood as a pressure for continuous quickness or change imposed on teachers. Rather, it should be considered as teachers’ perception of the school’s capacity to notice changing needs and respond to them appropriately.

In public schools, organizational agility should not be interpreted as the direct transfer of a private-sector performance logic into education. In schools, professional community, collegial sharing, collective learning, and collaboration among teachers are considered important for school functioning and improvement (Louis et al., 1996; Stoll et al., 2006). In the present study, agility is linked to teachers’ perceptions that the school can recognize problems in a timely manner, take teachers’ input into account, maintain communication, adapt resources and routines when necessary, and develop coordinated responses to changing conditions (Sharifi and Zhang, 1999; Overby et al., 2006; Teece et al., 2016). In this sense, organizational agility is not positioned as a characteristic that directly eliminates workplace loneliness. Rather, it is treated as a complementary organizational perception reflecting the extent to which teachers view school functioning as responsive, participatory, and capable of timely adjustment. This approach makes it possible to relate agility to teachers’ social and psychological experiences while remaining within the interpretive limits of a cross-sectional design.

## 3 Theoretical framework

The model proposed in this study was developed by considering the Job Demands–Resources model, Conservation of Resources theory, and the need to belong perspective together. The JD-R model suggests that employees’ work experiences can be better understood by considering both the demanding aspects of work and the resources available to employees. Job demands refer to work characteristics that require physical, cognitive, or emotional effort and may be associated with strain when they persist over time. Job resources, in contrast, refer to conditions that facilitate work, support the achievement of work goals, and may be associated with employee well-being (Demerouti et al., 2001; Bakker and Demerouti, 2017).

Within this framework, perceived organizational toxicity can be interpreted as a social and psychological job demand that may make teachers’ experiences of trust, open communication, professional sharing, and cooperation at school more difficult. This interpretation does not mean that toxicity directly causes loneliness. More cautiously, perceived toxicity is treated as an organizational condition that can be considered together with teachers’ relational experiences at school and their evaluations of how the school functions. In a toxic or psychologically unsafe environment, teachers may be less willing to share problems openly, contribute to information flow, or participate in joint problem-solving processes (Edmondson, 1999; Detert and Edmondson, 2011). This may also be related to lower perceptions of the school’s capacity to recognize changing needs and respond to them in a timely manner.

Organizational agility is positioned in this model as a contextual and functional school resource. In the present study, agility refers to teachers’ perceptions of the school’s capacity to notice changing conditions, develop appropriate responses to emerging needs, maintain communication and coordination, and adapt its functioning when necessary. Therefore, agility is not used only in the sense of quick decision-making or managerial flexibility. In the school context, it is considered more cautiously as teachers’ evaluation of the extent to which the school is responsive to problems and changing needs. Considering that professional community, collective learning, and collaboration among teachers are related to school functioning, perceptions of agility may also be considered together with the school’s capacity for information sharing and joint problem solving (Louis et al., 1996; Stoll et al., 2006).

At this point, it should be clarified that the present model does not apply the JD-R approach in a direct textbook sense. In the JD-R literature, job demands and job resources are often treated as co-existing conditions that explain employee experiences. In the present study, however, perceived organizational toxicity is proposed to be negatively associated with teachers’ perceptions of agile school functioning. In other words, the model examines a resource-depletion pathway in which perceived toxicity is associated with organizational agility as a functional school resource, and this pattern is considered together with workplace loneliness. Therefore, the model should not be interpreted as evidence of a causal resource-loss process. Rather, it should be understood as a theoretically grounded statistical mediation model tested with cross-sectional data.

Conservation of Resources theory offers a complementary explanation for this interpretation. According to this theory, individuals seek to obtain, protect, and maintain valued resources, and threats to or losses of these resources may be associated with unfavorable experiences (Hobfoll, 1989). In the school environment, trust, collegial support, open communication, information sharing, timely administrative response, and attention to teacher voice can be considered meaningful relational and functional resources for teachers. Perceived organizational toxicity may be associated with the weakening of these resources, whereas organizational agility may reflect teachers’ perceptions of the school’s capacity to sustain and mobilize such resources at the level of school functioning. However, because the study is cross-sectional, this relationship should be interpreted as a theoretically meaningful pattern of associations among variables rather than as a causal process.

The inclusion of workplace loneliness in the model is also consistent with the need to belong perspective. This perspective emphasizes that individuals need stable and meaningful interpersonal relationships and that this need is important for psychological functioning (Baumeister and Leary, 1995). For teachers, school is not only an institution in which work tasks are performed, but also a social environment in which professional identity, sharing, trust, and a sense of belonging are shaped. Therefore, teachers’ workplace loneliness should not be considered separately from the relational climate of the school and teachers’ perceptions of school functioning. By examining workplace loneliness together with perceived organizational toxicity and organizational agility, the present study aims to contribute to a more organizational understanding of teachers’ social and psychological experiences at school.

## 4 Hypotheses development

Organizational toxicity is discussed together with negative interaction patterns that may weaken the quality of relationships in the workplace. Toxic workplace environments are associated with disrespect, exclusion, humiliation, aggression, and similar behaviors that make constructive communication more difficult (Rasool et al., 2021; Wang et al., 2020). Lower-intensity negative behaviors may also become more wearing for workplace relationships when they are repeated or sustained through reciprocal reactions (Andersson and Pearson, 1999; Ghosh et al., 2011). In the school context, such an atmosphere may be related to how teachers share professional concerns, seek support, and communicate openly with colleagues. Given that workplace loneliness is connected with how employees experience social relationships in the workplace, teachers’ higher perceptions of organizational toxicity are expected to be associated with higher workplace loneliness (Wright et al., 2006).

*H1*: Perceived organizational toxicity is positively associated with workplace loneliness.

Organizational agility is related to an organization’s capacity to notice changing conditions and respond to emerging needs (Overby et al., 2006; Sharifi and Zhang, 1999). In the agility literature, this construct is explained through features such as flexibility, responsiveness, adaptability, and the ability to act appropriately under changing conditions (Asghar et al., 2026; Sherehiy et al., 2007). In contrast, in a school environment perceived as toxic, trust, open communication, and willingness to act together may become weaker. Therefore, as teachers’ perceptions of organizational toxicity increase, their perceptions of the school’s agile functioning are expected to be lower.

*H2*: Perceived organizational toxicity is negatively associated with organizational agility.

In this study, organizational agility refers to teachers’ perceptions of the school’s capacity to respond to emerging needs and adapt its functioning to changing conditions. Agility should not be viewed as a general characteristic that produces positive outcomes in all circumstances; the relationship between agility and organizational outcomes may vary depending on context (Bekos et al., 2025). At the same time, there is evidence that employees’ perceptions of organizational agility may be associated with work engagement and well-being (Ludviga and Kalvina, 2024). In the school context, teachers’ perceptions of the school as more agile may be considered together with more positive evaluations of communication, support, and problem-solving processes. Therefore, organizational agility is expected to be negatively associated with workplace loneliness.

*H3*: Organizational agility is negatively associated with workplace loneliness.

It is theoretically more plausible to expect organizational agility to play a partial rather than a full mediating role in this association. A toxic school environment may be associated with teachers’ workplace loneliness not only through weaker perceptions of the school’s agile functioning, but also through more direct relational experiences. Toxic or uncivil behavior patterns have been associated with negative interaction processes that may erode the quality of workplace relationships over time (Andersson and Pearson, 1999; Ghosh et al., 2011). The psychological safety literature also suggests that employees’ willingness to communicate openly, engage in learning behavior, and take interpersonal risks is related to the perceived safety of the social context in which they work (Edmondson, 1999; Newman et al., 2017; Frazier et al., 2017). Considered together, these two lines of literature suggest that perceived toxicity in schools may be associated with teachers’ experiences of sharing problems, seeking support, and feeling safe in professional relationships. For this reason, organizational agility is not treated as the only mechanism explaining the association between organizational toxicity and workplace loneliness, but as a contextual and functional school resource that may explain part of this association.

This interpretation can also be considered together with the Job Demands–Resources model and Conservation of Resources theory. According to the JD-R approach, employees’ work experiences can be better understood by considering job demands and available job resources together (Demerouti et al., 2001; Bakker and Demerouti, 2017). The model proposed in the present study does not apply the JD-R demand–resource distinction in a textbook sense. Rather, it suggests a resource-depletion pathway in which perceived toxicity, as a social and psychological demand, may be negatively associated with teachers’ perceptions of organizational agility as a functional school resource. Conservation of Resources theory also argues that individuals seek to obtain, protect, and maintain valued resources, and that threats to or losses of these resources may be associated with unfavorable work experiences (Hobfoll, 1989). Accordingly, organizational agility should not be interpreted as a causal mechanism that fully explains the association between perceived organizational toxicity and workplace loneliness. Instead, it is treated as a theoretically grounded statistical mediator that may explain part of this association. Because the study uses a cross-sectional design, this expectation should be interpreted as a cross-sectional statistical mediation model rather than evidence of a causal process.

*H4*: Organizational agility partially statistically mediates the cross-sectional association between perceived organizational toxicity and workplace loneliness.

## 5 Method

### 5.1 Research design and sample

This study used a cross-sectional correlational design to examine the associations among teachers’ perceived organizational toxicity, organizational agility, and workplace loneliness. In the proposed model, perceived organizational toxicity was treated as the independent variable, workplace loneliness as the dependent variable, and organizational agility as the statistical mediating variable. The model was tested as a cross-sectional statistical mediation model. Therefore, the paths in the model were interpreted as theoretically grounded associations among variables rather than as evidence of causal or temporal ordering.

The study group consisted of teachers working in public primary and lower-secondary schools located in the central district of Rize Province, Türkiye. Data were collected through convenience sampling. Teachers were included in the study if they were actively working in public primary or lower-secondary schools in the central district of Rize during the data collection period and volunteered to participate in the study. Forms from participants working outside the target school context, forms containing a high level of missing data, and forms showing clear response patterns that could reduce response quality, such as straight-lining, were excluded from the analysis.

Teachers were recruited from multiple schools. However, school names, identification codes, and school-specific participation counts were not recorded on the data-collection forms. Consequently, the responses could not be linked to particular schools, and the distribution of participants across schools could not be reconstructed. It was therefore not possible to calculate school-level intraclass correlation coefficients or design effects, or to estimate multilevel models or cluster-robust standard errors. The findings were accordingly interpreted as associations among teacher-level perceptions rather than as school-level organizational effects. The exact number of schools contributing participants was not retained in the analytical records.

After data screening, the analyses were conducted with 301 valid participants. Of the participants, 193 were female (64.1%) and 108 were male (35.9%). In terms of educational level, 267 teachers held an undergraduate degree (88.7%) and 34 held a graduate degree (11.3%). The sample included 105 classroom teachers (34.9%) and 196 subject-area teachers (65.1%). Regarding school type, 121 teachers worked in primary schools (40.2%) and 180 worked in lower-secondary schools (59.8%). In terms of professional seniority, 32 teachers had 1–5 years of experience (10.6%), 74 had 6–10 years of experience (24.6%), and 195 had 11 years or more of experience (64.8%). Regarding tenure at the current school, 157 teachers had worked at the same school for 1–5 years (52.2%), 79 for 6–10 years (26.2%), and 65 for 11 years or more (21.6%). The age distribution showed that 11 participants were aged 21–25 years (3.7%), 30 were aged 26–30 years (10.0%), 73 were aged 31–35 years (24.3%), 93 were aged 36–40 years (30.9%), and 94 were aged 41 years or older (31.2%).

The analyses were conducted using the 301 valid responses available after data screening. No universal participant-to-parameter rule was used to establish sample-size adequacy because sample-size requirements in structural equation modeling depend on model complexity, factor loadings, path coefficients, distributional characteristics, and the magnitude of the effects being examined (Wolf et al., 2013). Likewise, RMSEA-based power depends on the model degrees of freedom and the null and alternative RMSEA values specified (MacCallum et al., 1996), while power to detect an indirect association depends on the magnitudes of both component paths and the method used to evaluate the indirect effect (Fritz and MacKinnon, 2007). Accordingly, the achieved sample size was considered in relation to the relatively parsimonious measurement and structural models, but it should not be interpreted as demonstrating adequate statistical power for every possible small effect. Standard errors and bias-corrected bootstrap confidence intervals based on 5,000 resamples were therefore reported to convey the precision of the principal estimates.

### 5.2 Data collection procedure and ethical considerations

Data were collected between March and April 2024 after the required ethics committee approval had been obtained. The study was approved by the Social and Human Sciences Ethics Committee of Recep Tayyip Erdoğan University (Approval No. 2023/300; October 25, 2023). The researchers visited public primary and lower-secondary schools in the central district of Rize and approached teachers who met the inclusion criteria. The purpose and voluntary nature of the study were explained to the teachers, and those who agreed to participate completed printed, paper-and-pencil forms. Participation was not arranged through an online survey, and no school-level identifier was included on the forms.

Participants were informed about the purpose of the study, the voluntary nature of participation, their right to withdraw from participation at any time, and the fact that their responses would be used only for scientific purposes. No names, identity numbers, school names, or other information that could personally identify participants were requested. The forms were completed individually by the participants after voluntary consent had been obtained.

Approximately 350 teachers were reached during the study, and 322 forms were obtained. To ensure data quality, the forms were reviewed before analysis. Forms containing a high level of missing data, forms from outside the target sample, or forms showing clear response patterns that could reduce response quality were excluded from the analysis. As a result, 301 forms were found suitable for analysis. Considering that approximately 350 teachers were reached, the form return rate was approximately 92.0%, and the usable valid response rate was approximately 86.0%.

### 5.3 Measures

Data were collected using a personal information form and three self-report measures. The personal information form included questions concerning participants’ gender, educational level, teaching field, school type, professional seniority, tenure at the current school, and age group. Perceived organizational toxicity was assessed using the Perceived Organizational Toxicity Scale, organizational agility using the Organizational Agility Scale, and workplace loneliness using the Loneliness at Work Scale. For descriptive and correlational analyses, mean scores based on the items of each scale were used so that all study variables could be interpreted on the original 1–5 response metric.

#### 5.3.1 Perceived organizational toxicity

Perceived organizational toxicity was measured using the Perceived Organizational Toxicity Scale developed by Kasalak (2015). The scale consists of 16 items and four subdimensions: toxicity arising from narcissistic behaviors, toxicity arising from aggressive behaviors, toxicity arising from unethical behaviors, and toxicity arising from rigid behaviors. Each subdimension contains four items. The items were presented as statements completing the common introductory phrase “In my work environment….” An example item is “Condescending attitudes are displayed in my work environment.” Participants responded to the items on a five-point scale ranging from 1 = never to 5 = always. No items were reverse-scored. Higher scores indicate higher perceived organizational toxicity. For descriptive statistics and correlation analyses, an overall organizational toxicity score was calculated by averaging the 16 items. In the structural model, organizational toxicity was specified as a latent variable represented by the narcissistic, aggressive, unethical, and rigid behavior subdimension scores. Cronbach’s alpha for the scale in the present study was 0.941. The separate confirmatory factor analysis produced the following fit indices: χ^2^/df = 2.705, GFI = 0.905, CFI = 0.952, RMSEA = 0.075, and SRMR = 0.0415.

#### 5.3.2 Organizational agility

Organizational agility was measured using the Organizational Agility Scale developed by Sharifi and Zhang (1999) and adapted into Turkish by Akkaya and Tabak (2018). The final Turkish form consists of 17 items and four subdimensions. The competency subdimension contains eight items, whereas the flexibility, responsiveness, and quickness subdimensions each contain three items. During the Turkish adaptation process, three items from the initial 20-item form were removed because of insufficient factor loadings, resulting in the final 17-item, four-factor structure. The Turkish adaptation was originally developed for business organizations. In the present study, the organizational referent in the items was adapted to the educational context by replacing the expression corresponding to “our organization/business” with “our school.” An example item from the form administered in this study is “All work processes in our school are defined in a simple, clear, and explicit manner.” This wording adjustment was made to align the organizational referent with the school context; the five-point response format and the intended four-dimensional structure of the scale were retained. Participants responded to the items on a five-point scale ranging from 1 = never to 5 = always. No items were reverse-scored. Higher scores indicate higher perceived organizational agility of the school. For descriptive statistics and correlation analyses, an overall organizational agility score was calculated by averaging the 17 items. In the structural model, organizational agility was specified as a latent variable represented by the competency, flexibility, responsiveness, and quickness subdimension scores. Cronbach’s alpha for the scale in the present study was 0.961. The separate confirmatory factor analysis produced the following fit indices: χ^2^/df = 2.859, GFI = 0.882, CFI = 0.955, RMSEA = 0.079, and SRMR = 0.0385. Considered together, these indices provided general support for the four-dimensional structure in the present sample, although the GFI value was below 0.90.

#### 5.3.3 Workplace loneliness

Workplace loneliness was measured using the Loneliness at Work Scale developed by Wright et al. (2006) and adapted into Turkish by Doğan et al. (2009). Participants responded to each item on a five-point scale ranging from 1 = not at all appropriate to 5 = completely appropriate. The scale consists of the emotional deprivation and social companionship dimensions. The data file contained the final item scores after the positively worded items had been reverse-scored in accordance with the scoring procedure of the scale; therefore, no additional reverse scoring was applied. Emotional deprivation was calculated as the mean of Items 1–9, social companionship as the mean of Items 10–16, and overall workplace loneliness as the mean of all 16 items. Higher scores indicate greater workplace loneliness.

The hypotheses of the present study concerned overall workplace loneliness rather than the two dimensions separately. Therefore, the overall scale score was used in the descriptive and correlational analyses and was represented as a single-indicator latent variable in the measurement and structural models. Cronbach’s alpha for the overall scale was 0.903. To identify the single-indicator latent construct, the unstandardized loading of the indicator was fixed at 1.00. The observed variance of the overall workplace loneliness score was 0.1636. Using the reliability-based error-variance approach, the residual variance was calculated as 0.1636 × (1–0.903) = 0.0159 and was fixed at 0.0159. This specification allowed measurement error in the overall workplace loneliness score to be represented rather than assuming that the observed score was measured without error. The separate confirmatory factor analysis of the scale showed acceptable fit: χ^2^/df = 2.774, GFI = 0.900, CFI = 0.933, RMSEA = 0.077, and SRMR = 0.0687.

### 5.4 Data analysis

Before the main analyses, the data set was examined for missing values, erroneous responses, and response patterns that could reduce data quality. After the relevant forms had been excluded, the analyses were conducted with 301 participants. The distributional properties of the variables were evaluated using skewness and kurtosis values together with Q–Q plots. Skewness values ranged from −0.29 to 1.59, whereas kurtosis values ranged from −0.44 to 1.86. These values were considered to fall within commonly accepted limits for structural equation modeling studies (Tabachnick and Fidell, 2019; West et al., 1995).

To facilitate the descriptive interpretation of the mean scores, the 1–5 scale range was divided into three equal-width categories. The category width was calculated by dividing the difference between the maximum and minimum possible scores by the number of categories: (5–1) / 3 = 1.33. Accordingly, mean scores between 1.00 and 2.33 were described as low, scores between 2.34 and 3.66 as moderate, and scores between 3.67 and 5.00 as high. These categories were not normative, diagnostic, or empirically validated cutoff points established by the scale developers. They were used only as equal-width descriptive intervals to facilitate the presentation of the sample means. The terms low, moderate, and high should therefore be interpreted as descriptive labels rather than as absolute or diagnostic levels.

The associations among the study variables were initially examined using Pearson correlation analysis. To assess the possibility of multicollinearity, the bivariate correlations were reviewed. All correlations were below 0.85 in absolute value, indicating that the variables did not exhibit excessive overlap (Kline, 2016). A structural equation model was subsequently specified to examine the direct and indirect associations among perceived organizational toxicity, organizational agility, and workplace loneliness.

Demographic variables were not included as covariates in the structural model. The study was designed to examine the theoretically specified associations among perceived organizational toxicity, organizational agility, and workplace loneliness rather than to estimate demographic differences or causal effects adjusted for an exhaustive set of background characteristics. No *a priori* hypotheses identified gender, age, professional seniority, tenure at the current school, school type, teaching field, or educational level as confounders of the proposed associations. In addition, age, professional seniority, and tenure at the current school represent conceptually overlapping career-related characteristics, and their simultaneous inclusion could complicate interpretation without a clear theoretical basis. Therefore, demographic variables were reported descriptively but were not entered into the structural model solely on the basis of availability or *post hoc* statistical significance. This decision does not imply that demographic characteristics are irrelevant; rather, their possible conditional roles should be examined in studies specifically designed to test demographic differences or moderation.

Before the hypothesized structural paths were tested, a measurement model including the three study constructs was evaluated using maximum likelihood estimation. Perceived organizational toxicity was specified as a latent construct indicated by the narcissistic, aggressive, unethical, and rigid behavior subdimension scores. Organizational agility was specified as a latent construct indicated by the competency, flexibility, responsiveness, and quickness subdimension scores. These indicators were predefined subdimension scores derived from the established structures of the scales rather than *ad hoc* item parcels created for the present analysis. Their use provided a more parsimonious model while retaining the theoretically specified multidimensional organization of the constructs. Nevertheless, aggregation at the subdimension level may conceal item-level measurement problems and should not be interpreted as evidence that item parceling is generally preferable (Little et al., 2002).

Workplace loneliness was represented as a single-indicator latent construct based on the overall scale score because the hypotheses concerned overall workplace loneliness rather than its dimensions separately. Following the identification approach for single-indicator latent variables, the unstandardized loading of the indicator was fixed at 1.00. Its residual variance was calculated using the unrounded observed variance of the overall score and the internal consistency coefficient: 0.1636 × (1–0.903) = 0.0159. The residual variance was therefore fixed at 0.0159, and the latent variance was freely estimated (Bollen, 1989; Kline, 2016). This specification retained the theoretical focus on overall workplace loneliness while explicitly representing measurement error rather than treating the observed score as error-free.

Measurement-model fit was evaluated using chi-square, degrees of freedom, the χ^2^/df ratio, GFI, AGFI, NFI, RFI, IFI, TLI, CFI, RMR, RMSEA, the 90% confidence interval for RMSEA, and PCLOSE (Brown, 2015; Kline, 2016). Convergent validity for the multi-indicator constructs was evaluated using standardized factor loadings, composite reliability, and average variance extracted. Discriminant validity between the multi-indicator constructs was assessed using the Fornell–Larcker criterion (Fornell and Larcker, 1981). Because workplace loneliness was modeled using a single indicator, conventional multi-indicator composite reliability, AVE, Fornell–Larcker, and HTMT statistics were not calculated for this construct.

After the initial measurement model had been estimated, the modification indices were inspected. They indicated shared residual variance between the responsiveness and quickness indicators of organizational agility. Because these indicators represent conceptually related aspects of timely organizational response, one residual covariance was specified between them. This covariance had not been specified *a priori* and was therefore treated as a post hoc model respecification. No other residual covariances were added.

The hypothesized partial-mediation model was then estimated. The indirect association between perceived organizational toxicity and workplace loneliness through organizational agility was evaluated using 5,000 bootstrap resamples and bias-corrected 95% confidence intervals. An indirect association was considered statistically supported when its confidence interval did not include zero. To evaluate the robustness of the proposed structural specification, the hypothesized partial-mediation model was also compared with a full-mediation model, a no-mediation model, and a reverse-order alternative model. Nested models were compared using chi-square difference tests, whereas overall model fit and information criteria were also considered across the alternative specifications.

Because the principal study variables were collected from the same participants using self-report measures during the same data-collection period, the possibility of common method variance was examined through several complementary diagnostic procedures. First, Harman’s single-factor test was conducted using an unrotated exploratory factor analysis that included all scale items (Podsakoff et al., 2003). Second, tolerance and variance inflation factor values were examined as part of a full-collinearity assessment, using the VIF criterion of 3.3 proposed by Kock (2015). Third, a deliberately restrictive single-factor confirmatory factor model was estimated in which the four organizational toxicity dimensions, the four organizational agility dimensions, and the overall workplace loneliness score were specified as indicators of one common latent factor. In this model, the indicator residual variances were freely estimated, and no correlated residuals were specified. The fit of the single-factor model was compared descriptively with that of the proposed three-construct measurement model. These procedures were treated as diagnostic assessments rather than as definitive tests demonstrating the presence or absence of common method variance. Statistical analyses were conducted using SPSS 27.0 and AMOS 24.0.

## 6 Results

### 6.1 Descriptive statistics and correlations

Descriptive statistics for organizational toxicity, organizational agility, and workplace loneliness are presented in Table 1. In interpreting the scale scores, values between 1.00 and 2.33 were considered low, values between 2.34 and 3.66 were considered moderate, and values between 3.67 and 5.00 were considered high.

**Table 1:** Descriptive statistics for the study variables.

Variables	Min	Max	Mean	Std. deviation	Skewness	Kurtosis	Level
Organizational toxicity	1.00	3.19	1.34	0.49	1.590	1.866	Low
Organizational agility	1.94	5.00	3.91	0.72	−0.298	−0.448	High
Workplace loneliness	1.43	3.32	1.99	0.40	1.039	0.618	Low

As shown in Table 1, the mean organizational toxicity score fell within the descriptively defined low range. The mean organizational agility score fell within the descriptively defined high range, whereas the mean workplace loneliness score fell within the descriptively defined low range. Because these categories were based on equal-width intervals rather than validated normative cutoffs, they were used only to facilitate the descriptive interpretation of the sample means.

Pearson correlation analysis was conducted to examine the associations among the study variables. The correlation results are presented in Table 2.

**Table 2:** Correlations among the study variables.

Variables	Organizational toxicity	Organizational agility	Workplace loneliness
Organizational toxicity	1		
Organizational agility	−0.512^***^	1	
Workplace loneliness	0.528^***^	−0.435^***^	1

^***^p < 0.001.

As shown in Table 2, organizational toxicity was negatively and significantly associated with organizational agility (*r* = −0.512, *p* < 0.001). Organizational toxicity was positively and significantly associated with workplace loneliness (*r* = 0.528, *p* < 0.001). The association between organizational agility and workplace loneliness was negative and statistically significant (*r* = −0.435, *p* < 0.001). These findings show that the study variables were significantly related in the expected directions before testing the structural model.

### 6.2 Measurement model

**Figure 1:**
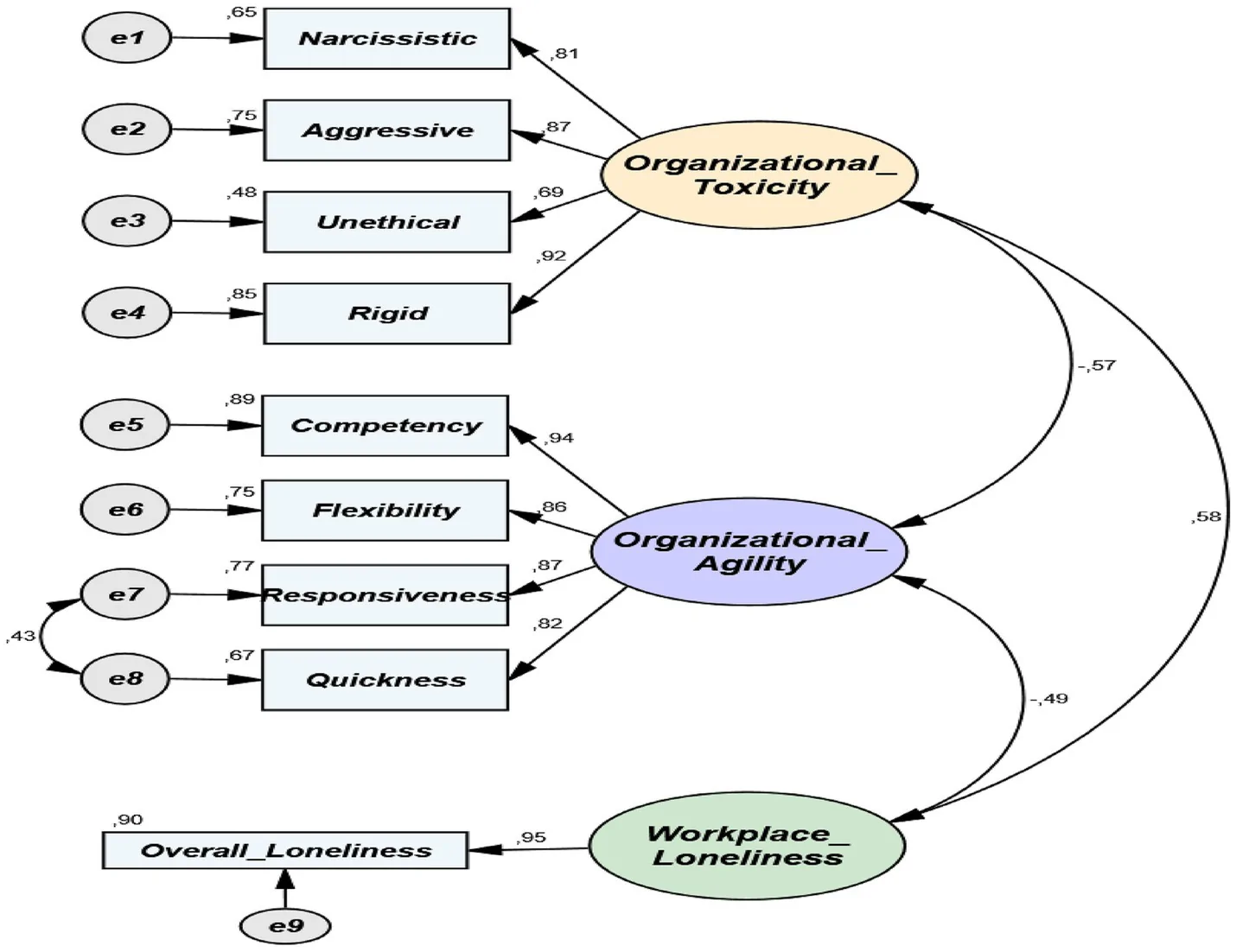
Standardized measurement model of perceived organizational toxicity, organizational agility, and workplace loneliness.

A three-construct measurement model was estimated before the hypothesized structural paths were examined. Perceived organizational toxicity and organizational agility were represented as latent constructs indicated by their respective subdimension scores. Workplace loneliness was represented as a single-indicator latent construct based on the overall scale score. The measurement model showed an acceptable-to-good overall fit to the data: χ^2^(24) = 64.837, *p* < 0.001, χ^2^/df = 2.702, GFI = 0.953, AGFI = 0.912, NFI = 0.969, RFI = 0.953, IFI = 0.980, TLI = 0.970, CFI = 0.980, SRMR = 0.050, and RMSEA = 0.075, 90% CI [0.054, 0.098]. The PCLOSE value was 0.029. Thus, although the incremental and absolute fit indices generally supported the model, the close-fit criterion was not supported, and the model was not characterized as demonstrating exact or uniformly excellent fit. The standardized measurement model is presented in Figure 1.

For perceived organizational toxicity, the standardized factor loadings ranged from 0.693 to 0.920. The loadings were 0.920 for rigid behaviors, 0.693 for unethical behaviors, 0.868 for aggressive behaviors, and 0.808 for narcissistic behaviors. The corresponding squared multiple correlations ranged from 0.481 to 0.846. Composite reliability was 0.895 and average variance extracted was 0.683, supporting the internal consistency and convergent validity of the construct.

For organizational agility, the standardized factor loadings ranged from 0.817 to 0.942. The loadings were 0.817 for quickness, 0.875 for responsiveness, 0.864 for flexibility, and 0.942 for competency. The corresponding squared multiple correlations ranged from 0.668 to 0.887. Composite reliability was 0.929 and average variance extracted was 0.767, supporting the internal consistency and convergent validity of the construct.

Workplace loneliness was modeled as a single-indicator latent construct. The unstandardized loading of the overall scale score was fixed at 1.00, and its reliability-based residual variance was fixed at 0.0159. The standardized loading was 0.950, and the squared multiple correlation was 0.902. Thus, by construction, 90.2% of the variance in the observed overall workplace loneliness score was represented by the latent workplace loneliness construct under the reliability-based specification. This value follows from the fixed error-variance approach and should not be interpreted as an independently estimated Cronbach’s alpha coefficient, composite reliability estimate, or conventional multi-indicator AVE value. The standardized and unstandardized measurement-model estimates for all three constructs are presented in Table 3.

**Table 3:** Standardized and unstandardized measurement-model estimates.

Construct and indicator	B	SE	C.R.	*p*	Standardized loading	SMC
Perceived organizational toxicity						
Rigid behaviors	1.000*	—	—	—	0.920	0.846
Unethical behaviors	0.702	0.049	14.439	<0.001	0.693	0.481
Aggressive behaviors	0.985	0.046	21.447	<0.001	0.868	0.753
Narcissistic behaviors	0.870	0.046	18.743	<0.001	0.808	0.653
Organizational agility						
Quickness	1.000*	—	—	—	0.817	0.668
Responsiveness	1.056	0.043	24.382	<0.001	0.875	0.765
Flexibility	1.095	0.061	17.970	<0.001	0.864	0.746
Competency	0.936	0.047	19.915	<0.001	0.942	0.887
Workplace loneliness						
Overall workplace loneliness score	1.000*	—	—	—	0.950	0.902

B, unstandardized estimate; SE, standard error; C.R., critical ratio; SMC, squared multiple correlation. *Fixed at 1.00 for model identification. The residual variance of the workplace loneliness indicator was fixed at 0.0159; AMOS displayed this value as 0.016 after rounding.

The latent correlations were negative between perceived organizational toxicity and organizational agility, *r* = −0.571, *p* < 0.001; positive between perceived organizational toxicity and workplace loneliness, *r* = 0.578, *p* < 0.001; and negative between organizational agility and workplace loneliness, *r* = −0.488, *p* < 0.001. For perceived organizational toxicity and organizational agility, the square roots of AVE were 0.827 and 0.876, respectively. Both values exceeded the absolute correlation between these two constructs, supporting their discriminant validity according to the Fornell–Larcker criterion (Fornell and Larcker, 1981). Because workplace loneliness was represented by a single indicator, conventional Fornell–Larcker and HTMT assessments were not conducted for that construct. The construct reliability and convergent-validity results are summarized in Table 4.

**Table 4:** Construct reliability and convergent validity.

Construct	Number of indicators	CR	AVE	√AVE
Perceived organizational toxicity	4	0.895	0.683	0.827
Organizational agility	4	0.929	0.767	0.876
Workplace loneliness	1	—	—	—

CR, composite reliability; AVE, average variance extracted. Workplace loneliness was modeled as a single-indicator latent construct; therefore, conventional multi-indicator CR, AVE, and square-root-of-AVE values were not calculated for this construct. The latent correlation between perceived organizational toxicity and organizational agility was −0.571. Because the square roots of AVE for both constructs exceeded the absolute value of this correlation, discriminant validity between the two multi-indicator constructs was supported.

Following inspection of the modification indices, a residual covariance was added between the responsiveness and quickness indicators. The covariance was positive and statistically significant, B = 0.081, SE = 0.016, C.R. = 5.073, *p* < 0.001, and the standardized residual correlation was 0.435. The covariance was considered theoretically plausible because responsiveness and quickness represent closely related aspects of timely organizational functioning. Nevertheless, because it was introduced after inspecting the modification indices, it was treated as a *post hoc* respecification that may be sample-specific and requires replication in independent samples.

### 6.3 Structural model and mediation analysis

After the measurement model had been evaluated, the hypothesized structural paths were estimated. Perceived organizational toxicity, organizational agility, and workplace loneliness were represented as latent constructs using the measurement specifications described in the preceding section. Workplace loneliness remained a single-indicator latent construct, with the unstandardized loading of the overall workplace loneliness score fixed at 1.00 and its reliability-based residual variance fixed at 0.0159. The post hoc residual covariance between the responsiveness and quickness indicators was retained in the structural model.

The structural model demonstrated an acceptable-to-good overall fit to the data: χ^2^(24) = 64.837, *p* < 0.001, χ^2^/df = 2.702, CFI = 0.980, TLI = 0.970, GFI = 0.953, AGFI = 0.912, and RMSEA = 0.075, 90% CI [0.054, 0.098]. The standardized root mean square residual was 0.050. Although the principal fit indices generally supported the model, the upper bound of the RMSEA confidence interval approached 0.10 and the PCLOSE value was 0.029. The model was therefore not characterized as showing uniformly excellent or close fit. Perceived organizational toxicity was negatively associated with organizational agility, B = −0.729, SE = 0.076, C.R. = − 9.636, *p* < 0.001, *β* = −0.571. Thus, higher perceived organizational toxicity was associated with lower perceived organizational agility. Organizational agility was negatively associated with workplace loneliness, B = −0.133, SE = 0.038, C.R. = − 3.536, *p* < 0.001, *β* = −0.234. Perceived organizational toxicity also retained a positive direct association with workplace loneliness after organizational agility was included in the model, B = 0.323, SE = 0.049, C.R. = 6.628, *p* < 0.001, *β* = 0.444.

**Figure 2:**
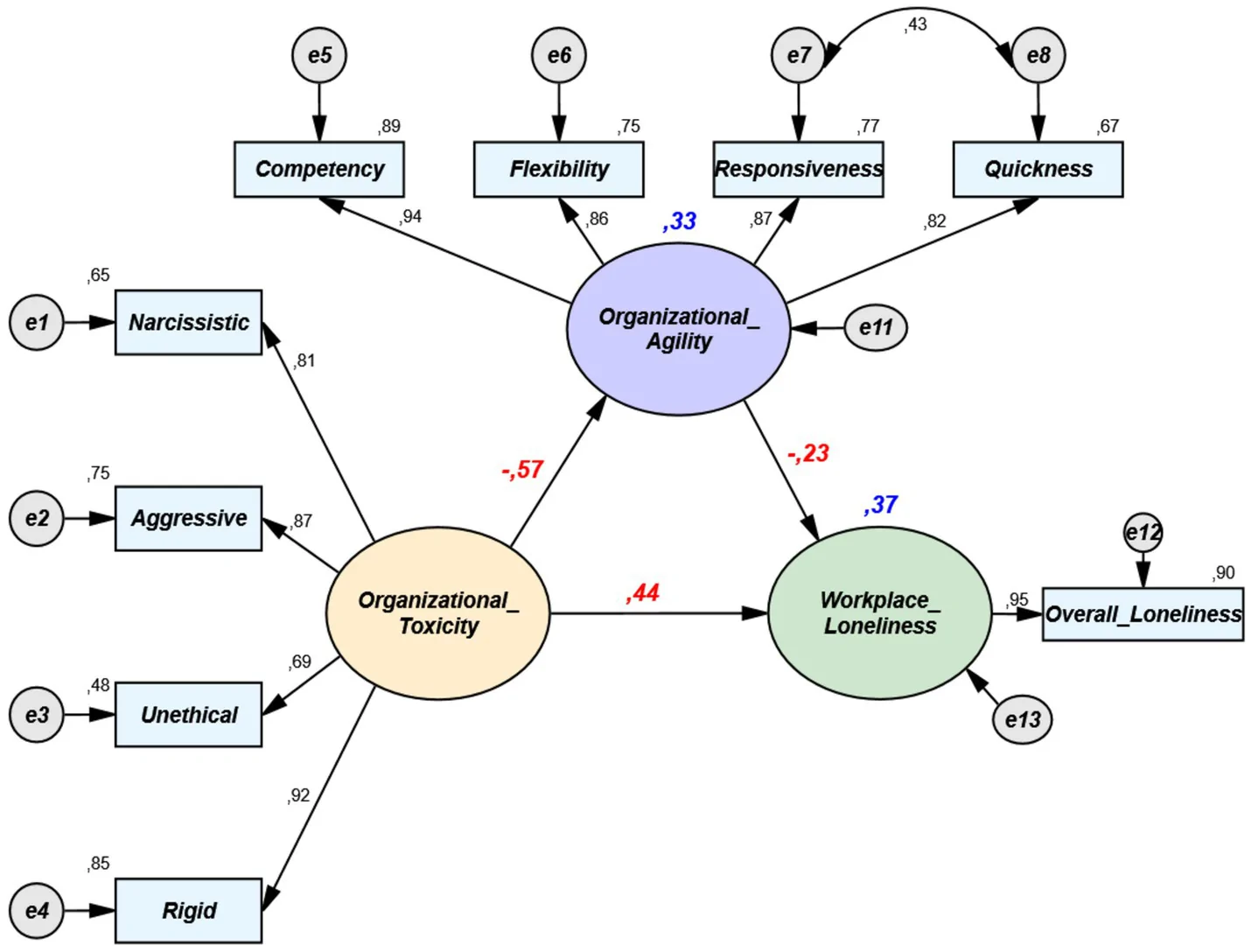
Standardized structural model of perceived organizational toxicity, organizational agility, and workplace loneliness.

Perceived organizational toxicity accounted for 32.6% of the variance in organizational agility (*R*^2^ = 0.326). Perceived organizational toxicity and organizational agility together accounted for 37.1% of the variance in workplace loneliness (*R*^2^ = 0.371). Although the model explained a meaningful proportion of the variance in workplace loneliness, a substantial proportion remained unexplained; therefore, the practical strength of the model should not be overstated. The standardized structural model is presented in Figure 2.

The direct, indirect, and total associations are summarized in Table 5.

**Table 5:** Direct, indirect, and total associations in the structural model.

Path	B	β	95% BC Bootstrap CI for B	*p*
Organizational Toxicity → Organizational Agility	−0.729	−0.571	[−0.901, −0.593]	<0.001
Organizational Agility → Workplace Loneliness	−0.133	−0.234	[−0.213, −0.058]	<0.001
Organizational Toxicity → Workplace Loneliness	0.323	0.444	[0.208, 0.439]	<0.001
Organizational Toxicity → Organizational Agility → Workplace Loneliness	0.097	0.134	[0.042, 0.164]	<0.001
Total association: Organizational Toxicity → Workplace Loneliness	0.420	0.578	[0.334, 0.523]	<0.001

B, unstandardized estimate; β, standardized estimate; BC, bias-corrected.

As shown in Table 5, the indirect association between perceived organizational toxicity and workplace loneliness through organizational agility was positive and statistically supported, B = 0.097, *β* = 0.134, 95% BC bootstrap CI [0.042, 0.164], *p* < 0.001. Because the confidence interval did not include zero, the indirect association was statistically different from zero. Perceived organizational toxicity was negatively associated with organizational agility, whereas organizational agility was negatively associated with workplace loneliness. Thus, higher perceived organizational toxicity was associated with lower perceived organizational agility, which in turn was associated with higher workplace loneliness.

The direct association between perceived organizational toxicity and workplace loneliness remained positive and statistically significant after organizational agility was included in the model, B = 0.323, *β* = 0.444, 95% BC bootstrap CI [0.208, 0.439], *p* < 0.001. The total association between perceived organizational toxicity and workplace loneliness was also positive and statistically significant, B = 0.420, *β* = 0.578, 95% BC bootstrap CI [0.334, 0.523], *p* < 0.001. These findings indicate that organizational agility statistically mediated part, but not all, of the cross-sectional association between perceived organizational toxicity and workplace loneliness. Accordingly, the results supported a pattern of partial statistical mediation rather than full mediation.

Taken together, H1 was supported because perceived organizational toxicity was positively associated with workplace loneliness. H2 was supported because perceived organizational toxicity was negatively associated with organizational agility. H3 was supported because organizational agility was negatively associated with workplace loneliness. H4 was also supported because the indirect association through organizational agility was statistically different from zero. Nevertheless, because the study used cross-sectional data, these findings should be interpreted as theoretically specified statistical associations rather than evidence of causal or temporal processes.

### 6.4 Alternative model tests

Three alternative structural specifications were examined to evaluate whether the hypothesized partial-mediation model provided a preferable representation of the observed covariance structure. The measurement specifications, the fixed residual variance of the single workplace-loneliness indicator, and the *post hoc* residual covariance between the responsiveness and quickness indicators were retained across all models. The fit statistics for the hypothesized and alternative models are summarized in Table 6.

**Table 6:** Comparison of the hypothesized and alternative structural models.

Model	χ^2^	df	χ^2^/df	CFI	TLI	RMSEA [90% CI]	AIC	BIC	ECVI
Hypothesized partial-mediation model	64.837	24	2.702	0.980	0.970	0.075 [0.054, 0.098]	106.837	184.687	0.356
Full-mediation model	106.806	25	4.272	0.960	0.943	0.104 [0.085, 0.125]	146.806	220.949	0.489
No-mediation model	77.240	25	3.090	0.975	0.963	0.083 [0.063, 0.105]	117.240	191.382	0.391
Reverse-order alternative model	64.837	24	2.702	0.980	0.970	0.075 [0.054, 0.098]	106.837	184.687	0.356

Compared with the hypothesized partial-mediation model, the full-mediation model showed Δχ^2^(1) = 41.969, *p* < 0.001, and the no-mediation model showed Δχ^2^(1) = 12.403, *p* < 0.001. The reverse-order model was observationally equivalent to the hypothesized model and produced identical global fit; this equivalence does not establish either temporal or causal ordering.

First, a full-mediation model was estimated by constraining the direct association between perceived organizational toxicity and workplace loneliness to zero. This model showed weaker fit than the hypothesized partial-mediation model: χ^2^(25) = 106.806, *p* < 0.001, χ^2^/df = 4.272, CFI = 0.960, TLI = 0.943, and RMSEA = 0.104, 90% CI [0.085, 0.125]. Its information criteria were also higher, AIC = 146.806, BIC = 220.949, and ECVI = 0.489. Although the indirect association remained statistically supported in the full-mediation model, B = 0.214, *β* = 0.294, 95% BC bootstrap CI [0.157, 0.282], *p* < 0.001, constraining the direct association to zero significantly worsened model fit relative to the partial-mediation model, Δχ^2^(1) = 41.969, *p* < 0.001. The full-mediation model explained 25.4% of the variance in workplace loneliness, compared with 37.1% in the partial-mediation model.

Second, a no-mediation model was estimated by constraining the association from organizational agility to workplace loneliness to zero while retaining the associations from perceived organizational toxicity to organizational agility and workplace loneliness. This model also showed weaker fit than the partial-mediation model: χ^2^(25) = 77.240, *p* < 0.001, χ^2^/df = 3.090, CFI = 0.975, TLI = 0.963, and RMSEA = 0.083, 90% CI [0.063, 0.105]. Its AIC, BIC, and ECVI values were 117.240, 191.382, and 0.391, respectively. Removing the organizational agility–workplace loneliness path produced a statistically significant deterioration in fit, Δχ^2^(1) = 12.403, *p* < 0.001. The no-mediation model explained 34.7% of the variance in workplace loneliness, compared with 37.1% in the partial-mediation model. These results supported retaining both the direct toxicity–loneliness association and the indirect association through organizational agility.

Third, a reverse-order alternative model was estimated in which workplace loneliness was associated with organizational agility, organizational agility was associated with perceived organizational toxicity, and workplace loneliness retained a direct association with perceived organizational toxicity. The reverse model produced exactly the same global fit statistics as the hypothesized partial-mediation model: χ^2^(24) = 64.837, p < 0.001, χ^2^/df = 2.702, CFI = 0.980, TLI = 0.970, and RMSEA = 0.075, 90% CI [0.054, 0.098]. Its AIC, BIC, and ECVI values were also identical to those of the hypothesized model.

Within the reverse specification, workplace loneliness was negatively associated with organizational agility, B = −0.856, SE = 0.105, C.R. = − 8.175, *p* < 0.001, *β* = −0.488. Organizational agility was negatively associated with perceived organizational toxicity, B = −0.297, SE = 0.047, C.R. = − 6.331, *p* < 0.001, *β* = −0.379, while workplace loneliness retained a positive direct association with perceived organizational toxicity, B = 0.540, SE = 0.081, C.R. = 6.657, *p* < 0.001, *β* = 0.393. The indirect association from workplace loneliness to perceived organizational toxicity through organizational agility was also statistically supported, B = 0.254, *β* = 0.185, 95% BC bootstrap CI [0.160, 0.380], *p* < 0.001.

The identical fit of the hypothesized and reverse-order models indicates that the cross-sectional covariance structure alone cannot distinguish between these alternative directional orderings. The hypothesized model was retained because its ordering followed the theoretical framework developed before the analyses; however, its superior causal or temporal validity cannot be inferred from model fit. Accordingly, the proposed indirect pathway should be interpreted as a theoretically specified cross-sectional statistical association rather than evidence that organizational toxicity temporally precedes organizational agility or workplace loneliness.

### 6.5 Common method variance assessment

Harman’s single-factor test showed that the first unrotated factor accounted for 35.79% of the total variance. Thus, the item covariance was not dominated by a single exploratory factor. In the full-collinearity assessment, tolerance values ranged from 0.721 to 0.811 and VIF values ranged from 1.233 to 1.386. All VIF values were below the 3.3 criterion proposed by Kock (2015), indicating no evident full-collinearity problem according to this diagnostic. A single-factor confirmatory model was also estimated using the four organizational toxicity dimensions, the four organizational agility dimensions, and the overall workplace loneliness score as indicators of one common latent factor. This model showed poor fit to the data: χ^2^(28) = 650.169, *p* < 0.001, χ^2^/df = 23.220, GFI = 0.605, AGFI = 0.365, NFI = 0.689, IFI = 0.698, TLI = 0.610, CFI = 0.697, and RMSEA = 0.272, 90% CI [0.254, 0.291], PCLOSE < 0.001. Its information criteria were also substantially higher, AIC = 684.169 and BIC = 747.190, than those of the proposed three-construct measurement model, AIC = 106.837 and BIC = 184.687. Taken together, these diagnostic results suggest that the covariance among the study measures was not adequately represented by one undifferentiated common factor and that no evident full-collinearity problem was detected. Nevertheless, these statistical procedures do not rule out common method variance. Because the principal constructs were measured through self-reports obtained from the same respondents at the same point in time, the possibility of method-related covariance was retained as a limitation when interpreting the findings.

## 7 Discussion

This study examined how teachers’ perceptions of organizational toxicity, organizational agility, and workplace loneliness were related in public primary and lower-secondary schools. Teachers who perceived their schools as more toxic also tended to perceive them as less agile and reported greater workplace loneliness, whereas stronger perceived organizational agility was associated with lower loneliness. Organizational agility accounted for part of the toxicity–loneliness association, but a substantial direct association remained, supporting partial statistical mediation. The descriptive findings should nevertheless be interpreted cautiously. Teachers reported descriptively low organizational toxicity and workplace loneliness and high organizational agility, but these labels were based on equal-width intervals rather than normative or diagnostic thresholds. The generally favorable profile may partly reflect voluntary participation, the characteristics of the participating teachers, or the limited geographical scope of the study. Even within this profile, sufficient variation remained for perceived toxicity and agility to be systematically associated with workplace loneliness.

The positive association between perceived organizational toxicity and workplace loneliness draws attention to the relational texture of everyday school life. Toxic work environments have been linked with disrespect, exclusion, humiliation, aggression, weakened support, and less favorable well-being-related experiences (Rasool et al., 2021; Wang et al., 2020). Repeated incivility, dismissive communication, and reciprocal negative reactions may gradually weaken trust and make workplace interactions more guarded (Andersson and Pearson, 1999; Ghosh et al., 2011). For teachers, this pattern may be experienced as hesitation to share professional difficulties, reluctance to seek support, or withdrawal from collegial conversations. This interpretation is consistent with the view that workplace loneliness concerns the perceived quality and adequacy of relationships rather than simply the number of people present (Wright et al., 2006). Teachers may interact frequently with students, colleagues, administrators, and parents while still feeling insufficiently understood, supported, or included. Previous research has connected teachers’ workplace loneliness with life satisfaction (Yılmaz and Arslan, 2013), while longitudinal evidence from broader employee samples suggests that workplace loneliness may also be associated with subsequent turnover (Sasaki et al., 2025). These findings support treating workplace loneliness as an organizationally relevant experience rather than as a private feeling detached from the workplace.

The negative association between perceived organizational toxicity and organizational agility suggests that the school’s relational climate and its perceived capacity to function responsively are intertwined. Organizational agility involves recognizing change and responding to emerging needs appropriately (Overby et al., 2006; Sharifi and Zhang, 1999) and should be understood as a multidimensional capacity rather than as speed alone (Asghar et al., 2026). In schools marked by distrust, defensive communication, or fear of negative reactions, important information may be withheld, problems may be discussed late, and coordinated action may become more difficult. This may help explain why stronger perceptions of toxicity coincided with weaker perceptions of agility. Organizational agility also remained negatively associated with workplace loneliness after perceived toxicity was taken into account. This finding is broadly consistent with research linking perceived strategic agility with employee engagement and well-being (Ludviga and Kalvina, 2024) and perceived organizational support with workplace loneliness (Tian et al., 2023). However, the study did not measure specific practices such as participatory decision-making, psychological safety, administrative support, or teacher voice. The agility coefficient should therefore be interpreted as an association with teachers’ overall perception of school functioning rather than as evidence that a particular administrative intervention reduces loneliness. Moreover, because the outcomes associated with agility may depend on complementary organizational capabilities and how change is managed (Bekos et al., 2025), this finding should not be generalized to every educational setting.

The mediation results sharpen this picture. The direct association between perceived organizational toxicity and workplace loneliness remained positive and statistically significant after organizational agility was included in the model (*β* = 0.444), whereas the standardized indirect association was smaller (*β* = 0.134). The total standardized association was 0.578, and perceived organizational toxicity and organizational agility together explained 37.1% of the variance in workplace loneliness. Organizational agility therefore accounted for a statistically supported but comparatively limited portion of the association, supporting partial rather than full statistical mediation. The remaining direct association may reflect interpersonal experiences such as humiliation, exclusion, distrust, or relational distancing, as well as unmeasured processes including psychological safety, perceived organizational support, trust, leadership behavior, and collegial climate. This pattern is compatible with, but does not directly test, the Job Demands–Resources model and Conservation of Resources theory. Perceived toxicity may be viewed as a social and psychological demand, whereas organizational agility may represent a contextual resource associated with the school’s capacity to respond and adapt (Bakker and Demerouti, 2017; Demerouti et al., 2001). Conservation of Resources theory similarly suggests that threats to valued resources may coincide with strain and less favorable work experiences (Hobfoll, 1989). The smaller indirect association indicates that agility represents only one part of a broader resource pathway. The findings are also consistent with the need-to-belong perspective, which emphasizes the importance of stable and meaningful relationships in understanding why feeling unheard, unsupported, or relationally distant may matter in teachers’ working lives (Baumeister and Leary, 1995).

The alternative-model tests place an important boundary around these interpretations. The full-mediation and no-mediation models fit the data less well than the proposed partial-mediation model, supporting the inclusion of both the direct association and the indirect association through organizational agility. However, the reverse-order model reproduced exactly the same global fit as the hypothesized model. The cross-sectional covariance structure therefore cannot determine whether perceived toxicity precedes lower agility and greater loneliness, whether teachers who feel lonely evaluate their schools as less agile and more toxic, or whether these perceptions influence one another reciprocally. Third variables such as negative affectivity, current stress, enduring pessimistic tendencies, or unmeasured personal and professional circumstances may also contribute to all three perceptions. Although the poor fit of the single-factor model indicated that the relationships could not be reduced to one undifferentiated common factor, this diagnostic result does not eliminate shared perception-based influences or other forms of common method variance. Longitudinal, multi-source, and multilevel designs are needed to distinguish among the hypothesized sequence, reverse associations, reciprocal processes, and common-cause explanations. Taken together, the findings connect teachers’ workplace loneliness with both the relational climate and perceived organizational functioning of schools, but they do not establish that toxicity causes loneliness or that agility protects teachers from it.

## 8 Theoretical implications

The findings invite a broader understanding of workplace loneliness. Loneliness at work is commonly discussed in relation to the perceived quality and adequacy of employees’ social relationships (Bryan et al., 2023; Wright et al., 2006). The present results suggest that it may also be connected with how employees experience the functioning of the organization itself—whether concerns are recognized, responses are coordinated, and changing needs are addressed. This pattern is relevant to the Job Demands–Resources model, in which demands and resources are generally treated as distinct features of the work environment that contribute to employee outcomes through different processes (Bakker and Demerouti, 2017; Demerouti et al., 2001). In the present model, perceived toxicity was associated with lower perceived organizational agility, which was in turn associated with greater workplace loneliness. This differs somewhat from a conventional JD-R formulation because it considers the possibility that a demanding relational environment may coincide with a weaker perception of an organizational resource. The findings therefore point to a possible resource-depletion pathway, although the cross-sectional design does not establish that toxicity temporally erodes agility.

This interpretation is also consistent with the interconnected view of resources proposed by Conservation of Resources theory (Hobfoll, 1989). Trust, open communication, collegial support, and confidence in the school’s capacity to respond may operate as a related set of relational and functional resources rather than as separate organizational conditions. Previous research has associated toxic workplace conditions with weaker support, lower trust, and less favorable employee experiences (Rasool et al., 2021; Wang et al., 2020). Within this broader resource pattern, perceived organizational agility may reflect whether teachers believe that available concerns and information can be translated into coordinated organizational action. The partial mediation result is especially informative here. Agility accounted for only part of the association between perceived toxicity and workplace loneliness, suggesting that functional response capacity represents one possible pathway rather than a complete explanation. Experiences such as exclusion, humiliation, distrust, or interpersonal distancing may remain relevant even when the school is otherwise perceived as responsive.

The results also give organizational agility a more explicitly relational meaning in educational settings. Agility has often been described in terms of recognizing change and responding with flexibility and speed (Overby et al., 2006; Sharifi and Zhang, 1999), while more recent work emphasizes the organizational processes, enabling mechanisms, and contextual conditions that make adaptive action possible (Asghar et al., 2026). For teachers, responsive school functioning may communicate more than organizational competence. It may also signal that their concerns are noticed, their input has value, and coordinated support is possible when difficulties arise. This interpretation brings agility into conversation with the need-to-belong perspective, which emphasizes the importance of stable and meaningful relationships (Baumeister and Leary, 1995). Agility does not replace relational concepts such as trust, psychological safety, or perceived organizational support. Rather, it may help explain whether relational support is reflected in the way the school responds and acts. This proposed connection between organizational responsiveness and professional belonging remains a theoretical interpretation that requires longitudinal, multilevel, and multi-source investigation.

## 9 Practical implications

The findings suggest that responses to teachers’ workplace loneliness should not focus exclusively on individual characteristics, social skills, motivation, or personal resilience. Such approaches may be useful, but they can leave the relational and organizational conditions of the school largely unexamined. School leaders may therefore benefit from viewing workplace loneliness as part of teachers’ broader experience of school climate, communication, and organizational functioning. One practical priority is to pay closer attention to the everyday interactions through which toxic perceptions may develop. Teachers need channels through which they can raise professional concerns without expecting ridicule, exclusion, retaliation, or prolonged silence. This does not necessarily require elaborate programs. Consistent communication, fair treatment, accessible leadership, and a willingness to address interpersonal tensions before they become entrenched may help create a safer professional environment. Administrators should also be attentive to less visible patterns, such as repeated dismissiveness, selective communication, or the gradual exclusion of particular teachers from professional exchanges. The findings also invite a more careful understanding of organizational agility in schools. Agility should not be equated simply with making decisions quickly. From a teacher’s perspective, responsive school functioning may also involve recognizing emerging needs, communicating clearly, following up on concerns, and adjusting practices when existing arrangements are no longer working well. Rapid decisions that are poorly explained or disconnected from teachers’ everyday work may be experienced as instability rather than agility. Timeliness is therefore likely to be most meaningful when it is accompanied by consistency, transparency, and sensitivity to the school context.

Teacher participation may be relevant within this process. When changes affect classroom practice, workload, coordination, or professional responsibilities, providing teachers with meaningful opportunities to express their views may make organizational responses better informed and more understandable. Participation should not be reduced to asking for opinions after decisions have effectively been finalized. Feedback processes are more credible when teachers can see how concerns were considered, why a particular course of action was chosen, and what follow-up will occur. Collegial support also deserves attention, although it should not be treated as a substitute for addressing harmful organizational conditions. Professional sharing meetings, peer-support arrangements, mentoring for teachers who are new to a school, and spaces where staff can discuss difficulties may create opportunities for connection. Their value, however, depends on how they are experienced. Formal activities are unlikely to be sufficient when teachers do not feel genuinely heard or when concerns raised in those settings are repeatedly left unresolved. Supportive initiatives need to be accompanied by trust, continuity, and visible organizational follow-through.

At the policy level, programs intended to support teacher well-being may be more useful when they consider the organization of school life alongside individual psychological support and professional development. Communication quality, administrative accessibility, fair procedures, teacher voice, and the school’s capacity to respond to changing needs may all form part of the context in which teachers feel recognized and supported. This broader perspective may help prevent workplace loneliness from being framed solely as a personal difficulty for teachers to manage on their own. These implications should nevertheless be interpreted within the limits of the evidence. The study measured teachers’ perceptions of organizational toxicity, organizational agility, and workplace loneliness; it did not evaluate particular leadership practices or test the effectiveness of communication programs, participatory decision-making, mentoring, or administrative-response interventions. The practices outlined above are therefore theory-consistent and evidence-informed suggestions rather than interventions demonstrated to reduce workplace loneliness by the present study. Their effectiveness should be assessed through longitudinal, experimental, or intervention-based research in different school settings.

## 10 Limitations and future research

The findings should be considered in light of the study’s cross-sectional design. All variables were measured at one point in time, so the proposed sequence from perceived organizational toxicity to organizational agility and workplace loneliness cannot be interpreted as a temporal or causal process. This limitation is especially important because the reverse-order model reproduced the same overall fit as the hypothesized model. Teachers who feel lonely at work may evaluate their schools as less agile and more toxic, the relationships may operate reciprocally, or all three perceptions may change together over time. Longitudinal research with repeated measurements would provide a stronger basis for examining temporal ordering and possible reciprocal relationships. The reliance on self-report data presents a related limitation. Perceived toxicity, agility, and loneliness are subjective experiences, making teachers’ own reports appropriate and theoretically meaningful. At the same time, collecting all principal variables from the same respondents during the same data-collection period creates a risk of shared method variance. Harman’s single-factor test, the full-collinearity assessment, and the single-factor confirmatory model did not suggest that the observed covariance could be adequately reduced to one common factor. These checks are diagnostic rather than conclusive, however, and cannot rule out all forms of common method variance (Podsakoff et al., 2003). Stable response tendencies, negative affectivity, current occupational stress, or other unmeasured personal circumstances could also influence how teachers evaluated all three constructs. Future research could reduce this uncertainty by combining teacher reports with administrator or colleague assessments, school-level records, temporally separated measurements, and theoretically appropriate marker variables.

The sampling context also limits the reach of the findings. Participants were teachers working in public primary and lower-secondary schools in the central district of one province in Türkiye and were recruited through convenience sampling. The results should therefore not be assumed to represent teachers in other provinces, rural communities, private schools, upper-secondary institutions, or education systems with different administrative structures. Replications across regions, school levels, and national settings would help clarify which parts of the observed pattern are context-specific and which are more broadly reproducible. Teachers were recruited from multiple schools, but school identifiers and school-specific participation counts were not recorded. The possible dependence of responses from teachers working in the same school could therefore not be evaluated using intraclass correlations or design effects, and cluster-robust or multilevel analyses could not be conducted. If within-school dependence was present, the conventional standard errors may not fully reflect the nested structure of the data. Future research should retain anonymized school codes and recruit sufficient numbers of schools and teachers within schools to permit multilevel structural equation modeling. The measurement strategy should also be kept in view. Organizational toxicity and organizational agility were represented by theoretically defined subdimension indicators, whereas workplace loneliness was modeled as a single-indicator latent construct based on the overall scale score. Fixing the loading and reliability-based residual variance allowed measurement error to be represented while preserving the study’s focus on overall loneliness. Even so, this approach does not provide the same level of measurement detail as an item-level or multidimensional latent model, and its estimates depend partly on the reliability value used to fix the residual variance. Studies with larger and more diverse samples could compare item-level, multidimensional, and single-indicator specifications and examine whether emotional deprivation and social companionship show different organizational associations.

One residual covariance between the responsiveness and quickness indicators of organizational agility was added after the modification indices had been examined. Although the covariance was conceptually plausible and was reported as a *post hoc* respecification, it remains sample-informed. Its replicability should therefore be assessed in an independent sample rather than assumed from the present results. Organizational agility explained part, but not all, of the association between perceived organizational toxicity and workplace loneliness. Other processes may be equally or more important, including psychological safety, perceived organizational support, organizational trust, leadership behavior, collegial support, teacher voice, and professional belonging. Future studies could examine these constructs as parallel or sequential mediators rather than assuming that agility represents the only pathway. Qualitative research may also reveal how teachers distinguish between a school that is genuinely responsive and one that merely introduces rapid or frequent changes.

Demographic characteristics were reported descriptively but were not entered into the structural model because no *a priori* hypotheses identified them as confounders of the proposed associations. This decision avoids selecting covariates solely because they were available or statistically significant after inspection, but it also means that possible variation across demographic and professional groups was not examined. Theory-driven multigroup or moderation studies could investigate whether the relationships differ by career stage, school type, teaching field, or other professionally relevant characteristics. These limitations do not negate the observed associations, but they define what can reasonably be concluded from them. The present findings offer a cross-sectional account of how teachers’ perceptions of relational climate, organizational responsiveness, and workplace loneliness are connected. Establishing how these experiences develop, influence one another, and vary across schools will require longitudinal, multilevel, and multi-source evidence.

## 11 Conclusion

This study examined how teachers’ perceptions of organizational toxicity, organizational agility, and workplace loneliness were connected in public primary and lower-secondary schools. Teachers who viewed their schools as more toxic also tended to view them as less agile and to report greater loneliness at work. Perceived organizational agility was, in turn, associated with lower workplace loneliness and accounted for a modest part of the relationship between toxicity and loneliness. The persistence of a comparatively strong direct association indicates that organizational agility does not provide a complete explanation for why toxicity and workplace loneliness are related. Harmful interpersonal experiences, weakened trust, exclusion, and other unmeasured organizational processes may also be involved. The findings therefore support partial statistical mediation rather than a model in which agility fully carries the association.

The study also highlights a broader way of thinking about workplace loneliness among teachers. Loneliness at school may not be understood adequately as an individual difficulty or as a simple absence of social contact. It may also be connected with how teachers experience the relational climate of the school and whether the organization appears capable of recognizing concerns, coordinating action, and responding when difficulties arise. In this sense, organizational agility may have relational significance as well as functional significance. These conclusions remain bounded by the cross-sectional and self-report nature of the evidence. The reverse-order model produced the same overall fit as the hypothesized model, showing that the data cannot establish whether perceived toxicity precedes lower agility and greater loneliness, whether loneliness shapes organizational perceptions, or whether the relationships are reciprocal. The results should therefore be read as a coherent pattern of association rather than as evidence of causal effects. Despite these limitations, the study brings together relational climate, organizational responsiveness, and workplace loneliness within a single framework and identifies organizational agility as one possible link between them. Future longitudinal, multilevel, and multi-source research can build on this framework to examine how these experiences develop over time and whether changes in school conditions are followed by changes in teachers’ sense of connection at work.

## Data Availability

The datasets generated and analyzed during the current study are not publicly available because they contain participant-level survey data, but they are available from the corresponding author upon reasonable request. Requests to access these datasets should be directed to gokhan.kahveci@erdogan.edu.tr.
