# Correction: Perceived organizational toxicity and workplace loneliness among public-school teachers in one province of Türkiye: a cross-sectional statistical mediation analysis of organizational agility

**DOI:** 10.3389/fpsyg.2026.2031998

**Published:** 2026-10-28

**Authors:** Ahmet Mesut Karadağ, Gökhan Kahveci

**Affiliations:** 1 Department of Educational Sciences, Institute of Graduate Education, Recep Tayyip Erdogan University, Rize, Türkiye; 2 Department of Educational Sciences, Faculty of Education, Recep Tayyip Erdogan University, Rize, Türkiye

**Keywords:** mediation, organizational agility, organizational psychology, organizational toxicity, school climate, teachers, Türkiye, workplace loneliness

In the published article, the following statement regarding the origin of the article was inadvertently omitted from the **Acknowledgment**:

“This article is based on the first author's master's thesis, completed under the supervision of the second author.”

The correct **Acknowledgments** reads below:

The authors would like to thank the teachers who voluntarily participated in this study. This article is based on the first author's master's thesis, completed under the supervision of the second author.

The original version of this article has been updated.

